# Impact of Nutrition on Embryo Production in Cattle: Mechanistic Insights

**DOI:** 10.3390/ani16060892

**Published:** 2026-03-12

**Authors:** Ramanathan Kasimanickam, Vanmathy Kasimanickam

**Affiliations:** College of Veterinary Medicine, Washington State University, Pullman, WA 99164, USA

**Keywords:** energy balance, micronutrients, assisted reproduction, oocyte competence, embryo yield, developmental programming

## Abstract

Reproductive efficiency in cattle is critically dependent on embryo quantity and quality, particularly in assisted reproductive technology (ART) programs such as superovulation, embryo transfer, and embryo production. Nutrition is a key determinant of embryo yield through its regulatory effects on metabolic signaling, ovarian function, oocyte competence, and early embryogenesis. This review synthesizes the current evidence describing mechanistic links between nutritional status and embryo production in dairy and beef cattle across both in vivo and in vitro systems. Energy balance, protein supply, micronutrients, and fatty acids influence metabolic hormones including insulin, insulin-like growth factor-1, and leptin, which regulate hypothalamic–pituitary–gonadal axis activity, follicular recruitment, and steroidogenesis. Negative energy balance disrupts endocrine signaling, elevates circulating non-esterified fatty acids, increases oxidative stress, and impairs oocyte mitochondrial function, resulting in reduced embryo yield, compromised blastocyst quality, and diminished cryotolerance. Targeted micronutrients such as selenium, zinc, vitamins A and E, B-complex vitamins, and omega-3 fatty acids enhance antioxidant capacity, membrane integrity, and epigenetic regulation, thereby supporting embryo viability and post-transfer survival. Furthermore, early-life nutrition programs long-term reproductive capacity by influencing ovarian reserve establishment and oocyte epigenetic competence. Strategic nutritional management is therefore essential to optimize ART outcomes and promote sustainable genetic progress in cattle production systems.

## Simple Summary

Reproductive success in cattle, especially when using technologies like superovulation, embryo transfer, and in vitro embryo production, depends greatly on the number and quality of embryos produced. Nutrition is one of the most important factors influencing these outcomes. Proper feeding supports hormone balance and healthy ovarian function, which are necessary for producing high-quality oocytes and viable embryos. When cows experience poor energy balance, such as during early lactation, they may produce fewer and lower-quality embryos due to metabolic stress and increased fat breakdown. Adequate protein, vitamins, and minerals, such as selenium, zinc, and vitamins A and E, help to protect cells from damage and support normal embryo development. Beneficial fats, including omega-3 fatty acids, also contribute to improved embryo survival. In addition, nutrition during early life can influence a heifer’s future reproductive performance. Overall, consistent, well-balanced feeding programs are essential for maximizing the success of reproductive technology and supporting long-term herd productivity and genetic improvement.

## 1. Introduction

Assisted reproductive technologies (ARTs) have become central components of modern cattle breeding programs, facilitating accelerated genetic progress through superovulation, embryo transfer (ET), and embryo production. Although ARTs are frequently promoted as strategies to enhance reproductive efficiency, their outcomes are fundamentally constrained by the inherent reproductive competence of donor and recipient animals. Reproductive efficiency, encompassing estrous cyclicity, follicular dynamics, oocyte developmental competence, uterine environment, and embryo survival, remains the cornerstone of productivity and profitability in both dairy and beef systems. Within ART programs, its significance is further amplified, as these technologies depend upon and intensify existing physiological capacity rather than compensate for its deficiencies. Donor females exhibiting optimal metabolic balance, endocrine stability, and overall health demonstrate improved responsiveness to gonadotropin stimulation, greater embryo yield, and enhanced pregnancy success following transfer. Consequently, a comprehensive understanding of the biological and management determinants of reproductive efficiency is critical to maximizing the efficacy and long-term sustainability of ART in cattle production systems [1,2].

Success in ART programs depends not only on precise hormonal protocols, donor selection, and embryo handling, but also on the nutritional and metabolic status of the donor cow or heifer, which profoundly affects follicular dynamics, oocyte developmental competence, and embryonic survival [3,4]. In modern high-producing cattle, nutritional deficits or metabolic imbalances are often subtle yet sufficient to significantly reduce embryo yield, compromise blastocyst quality, and diminish post-transfer pregnancy rates [1,5].

The production of viable embryos relies on two interdependent factors: (1) quantity, the number of ovulated follicles and embryos available for collection, and (2) quality, the developmental competence, cytoplasmic and nuclear maturation, and genetic integrity of the oocytes and resulting embryos [6,7]. These outcomes are tightly regulated by metabolic, hormonal, and cellular signaling pathways that are, in turn, influenced by nutrient availability [1,8]. Nutritional deficiencies, excesses, or imbalances can alter hypothalamic-pituitary-gonadal (HPG) axis activity, disrupt steroidogenesis, impair granulosa and theca cell function, and compromise intra-follicular microenvironments [1,9,10]. Such perturbations reduce the number of viable oocytes capable of reaching the blastocyst stage and surviving cryopreservation or ET [11,12].

Energy balance reflects the relationship between dietary energy intake and energy expenditure. For example, negative energy balance (NEB) occurs when energy output exceeds intake, resulting in mobilization of body reserves. Energy balance plays a particularly critical role in reproduction. Negative energy balance, often observed in early lactation dairy cows or underfed heifers, reduces circulating insulin, insulin-like growth factor-1 (IGF-1), and leptin, which are essential for gonadotropin releasing hormone (GnRH) pulsatility, follicular recruitment, and oocyte competence [13,14]. Protein and amino acid supply modulate nitrogen balance and intracellular substrates necessary for cytoplasmic maturation, while fatty acid composition in the diet influences membrane fluidity, prostaglandin synthesis, and mitochondrial function within oocytes [15,16]. Micronutrients, including selenium, zinc, copper, manganese, and antioxidant vitamins, protect oocytes from reactive oxygen species (ROS)-induced damage, support steroidogenic enzyme function, and enhance blastocyst survival [17].

The timing of nutritional adequacy is also critical. Early-life nutrition during the prepubertal period can program the reproductive axis and ovarian reserve, affecting the number of primordial follicles, the size of the antral follicle pool, and the efficiency of later superovulation. Nutritional insults during this developmental window can induce epigenetic modifications that persist into adulthood, influencing oocyte competence, embryo quality, and even potentially transgenerational reproductive performance [18,19].

Bovine embryos can be produced through two primary pathways: (1) in vivo embryo production (superovulation followed by artificial insemination and embryo collection) and (2) in vitro embryo production (IVP), which includes oocyte retrieval, in vitro maturation (IVM), in vitro fertilization (IVF), and in vitro culture (IVC). While both systems are influenced by donor nutrition, the mechanisms differ. In vivo systems rely heavily on systemic endocrine and metabolic regulation of follicular dynamics and uterine environment, whereas IVP outcomes are influenced both by donor metabolic status and by culture conditions. This review distinguishes nutritional effects on these two embryo production systems, with primary emphasis on donor-mediated mechanisms relevant to superovulation and IVP programs.

This review explores the multifaceted impact of nutrition on embryo yield, integrating endocrine, metabolic, molecular, and ART-specific perspectives. It examines the roles of macronutrients, micronutrients, fatty acids, and body condition in modulating folliculogenesis, oocyte competence, and embryo development. Mechanisms by which energy balance and early-life nutrition influence reproductive performance are highlighted, alongside practical strategies for optimizing donor nutrition in ART programs [5,20]. Finally, the economic implications of nutritional optimization in maximizing embryo yield, improving post-transfer success, and enhancing lifetime herd productivity are considered [1]. By providing a comprehensive, mechanistically grounded review, this discussion is designed to equip embryologists and veterinarians with actionable insights to integrate nutrition as a critical component of advanced reproductive management.

### Literature Scope and Selection Approach

This review was conducted as a narrative synthesis of the peer-reviewed literature examining the mechanistic relationships between nutrition and embryo technologies in cattle. Literature was identified through searches of PubMed, Web of Science, and Scopus databases covering the period 2000–2025, with particular emphasis on studies published within the past five years. Search terms included combinations of: “cattle,” “bovine,” “embryo production,” “superovulation,” “oocyte competence,” “negative energy balance,” “micronutrients,” “fatty acids,” “developmental programming,” “metabolomics,” and “epigenetics.” Only peer-reviewed original research articles and high-quality reviews were included. Conference abstracts were excluded unless supported by subsequent full publication. Studies were selected based on mechanistic relevance, experimental rigor, and applicability to in vivo or in vitro embryo technologies.

## 2. Nutrition and Reproductive Physiology in Cattle

Reproductive physiology in cattle is highly sensitive to nutritional status, as the HPG axis integrates metabolic cues to regulate follicular development, oocyte maturation, and overall fertility [21]. It is important to distinguish between nutrition and energy. Nutrition encompasses the intake and utilization of macro- and micro-nutrients that support structural, enzymatic, and regulatory functions. Energy represents the metabolic outcome of nutrient oxidation. Thus, energy balance is a consequence of nutritional status rather than a parallel entity. Throughout this review, energy balance is discussed as a functional expression of nutrient adequacy. In the context of ART, subtle variations in energy, protein, fatty acids, and micronutrients can profoundly influence ovarian responsiveness, oocyte competence, and embryo yield, making nutritional optimization a critical determinant of ART success [22,23]. Understanding the interplay between nutrition and reproductive physiology is essential for maximizing donor performance and embryo quality [21].

### 2.1. Macronutrients

#### 2.1.1. Energy Balance and Endocrine Modulation

Energy availability is the primary determinant of reproductive capacity. Negative energy balance, common in early lactation dairy cows or underfed heifers, leads to reductions in circulating insulin, IGF-1, and leptin, which are key metabolic signals regulating GnRH pulsatility and subsequent follicle stimulating hormone (FSH) and luteinizing hormone (LH) release [21,22]. Low insulin and IGF-1 impair granulosa cell proliferation and estradiol production, reducing antral follicle numbers and compromising oocyte cytoplasmic and nuclear maturation [22,24]. Conversely, positive energy balance supports robust folliculogenesis, enhances ovulatory response to exogenous gonadotropins, and improves blastocyst formation [22]. In ART programs, managing energy balance is crucial during superovulation and follicular growth to optimize the number and quality of oocytes retrieved [23].

#### 2.1.2. Protein and Amino Acid Influence

Protein intake modulates nitrogen availability, amino acid substrates, and overall metabolic efficiency, all of which are critical for reproductive function [1,24]. Excessive degradable protein increases blood urea nitrogen (BUN), altering intra-follicular pH and uterine environment, which negatively affect oocyte maturation and early embryo survival [1,24]. Adequate high-quality protein ensures sufficient essential amino acids for mitochondrial function, cytoplasmic organelle development, and epigenetic programming during oocyte maturation, directly impacting blastocyst quality and cryotolerance [1].

#### 2.1.3. Carbohydrate Metabolism and Reproductive Function

Carbohydrates are crucial in cattle diets because ruminal fermentation of structural and non-structural carbohydrates produces short-chain fatty acids, particularly propionate, which serves as the main gluconeogenic precursor for hepatic glucose production and supports energy balance critical for ovarian function and early embryo development [25]. Glucose availability influences insulin secretion, IGF-1 production, and ovarian steroidogenesis. Excess rapidly fermentable carbohydrates may induce subacute ruminal acidosis, systemic inflammation, and impaired follicular microenvironments. Conversely, inadequate carbohydrate intake limits glucose supply, reducing ATP availability within oocytes and compromising cytoplasmic maturation.

Adequate fermentable carbohydrate intake helps maintain circulating glucose and supports insulin and IGF-1 dynamics that are associated with follicular growth and reproductive hormone regulation in dairy cows [26]. Excessive insulin resistance and ketosis can mutually reinforce each other in dairy cows [27], which can reduce oocyte quality and consequently impair reproductive performance [28], thereby limiting the productive lifespan of cows. Diets deficient in glucogenic precursors can compromise energy status and delay postpartum ovarian activity, whereas excessive rapidly fermentable carbohydrates increase the risk of subacute ruminal acidosis (SARA), leading to dysbiosis, systemic inflammation, and endotoxin release that have been linked to disruptions in ovarian cyclicity and fertility outcomes [29]. Balanced carbohydrate nutrition with appropriate starch and fiber levels enhances energy status, supports estrogen synthesis, and improves ovulatory responses, ultimately benefiting reproductive efficiency in cattle.

#### 2.1.4. Water Intake and Reproductive Function

Water is an often overlooked yet essential macronutrient. Adequate hydration supports rumen fermentation, nutrient absorption, thermoregulation, and plasma volume maintenance. Dehydration reduces feed intake, alters metabolic hormone concentrations, and may indirectly impair follicular development and embryo yield. In high-producing dairy donors, optimal water intake is critical for maintaining metabolic stability during superovulatory cycles.

Water is essential for maintaining homeostasis, nutrient transport, and blood volume in dairy cattle, all of which support reproductive processes and oocyte quality. Inadequate water intake reduces dry matter intake and alters metabolic and endocrine profiles, which can impair follicular development and delay ovulation in lactating cows—a key determinant of oocyte competence [30]. Continuous access to clean, potable water influences physiological status and general health, indirectly affecting reproductive hormone balance and the follicular microenvironment needed for optimal oocyte maturation [31,32]. Water restriction has been shown to shift blood metabolites and stress responses in dairy cows, which may compromise the fluid and metabolic milieu around developing oocytes. Moreover, water quality issues can negatively affect overall metabolic efficiency and reproductive performance [33]. Ensuring adequate quantity and quality of drinking water helps stabilize metabolic and endocrine status, thereby supporting follicular development, oocyte viability, and subsequent embryo competence in dairy reproduction.

#### 2.1.5. Fatty Acids and Lipid Signaling

Fatty acid composition in the diet has direct consequences for oocyte competence and embryo development [23,34]. Omega-3 polyunsaturated fatty acids (PUFAs), such as EPA and DHA, enhance mitochondrial efficiency, improve follicular fluid composition, and modulate prostaglandin synthesis, which is critical for luteal support and uterine receptivity [6]. Balanced omega-6:omega-3 ratios prevent pro-inflammatory cascades that can impair early embryogenesis [35]. Lipid availability also influences cell membrane fluidity, organelle function, and signaling pathways within the oocyte and early embryo, making fatty acids an essential consideration for ART optimization [34].

### 2.2. Micronutrients

#### 2.2.1. Micronutrients and Antioxidant Protection

Trace minerals and vitamins serve as co-factors for steroidogenic enzymes and antioxidants that protect oocytes and embryos from reactive oxygen species (ROS) [35]. Selenium, zinc, copper, and manganese enhance granulosa and theca cell function, steroidogenesis, and DNA synthesis, while vitamins A, E, and the B-complex support cytoplasmic maturation, epigenetic regulation, and oxidative defense [26]. In metabolically stressed donors, antioxidant supplementation can markedly improve blastocyst formation rates and post-transfer viability, highlighting the importance of integrating micronutrients into ART protocols [35].

#### 2.2.2. Vitamins

Vitamins are essential regulators of oocyte and embryo development.

- **Vitamin A** plays a pivotal role in granulosa cell differentiation, steroidogenic enzyme expression, and oocyte cytoplasmic maturation. Retinoic acid, the bioactive form of vitamin A, modulates gene transcription during oogenesis and early embryogenesis. Deficiency impairs cumulus expansion, reduces meiotic competence, and negatively affects blastocyst development, while adequate supplementation enhances oocyte quality and embryo survival [8,36].
- **Vitamin E** serves as a lipid-soluble antioxidant, protecting oocyte and embryo membranes from ROS-mediated damage. Elevated oxidative stress, common in metabolically challenged high-producing cows, can impair mitochondrial function, compromise spindle integrity, and reduce cleavage and blastocyst formation rates. Vitamin E, particularly in synergy with selenium, enhances blastocyst viability, cryotolerance, and post-transfer pregnancy success [37,38].
- **B-complex vitamins**, including biotin and folate, support DNA synthesis, methylation reactions, and epigenetic programming in oocytes and early embryos. Folate deficiency has been associated with impaired blastocyst development and increased apoptotic activity within the inner cell mass. Supplementation ensures adequate nucleotide and methyl donor availability, facilitating proper cell division, differentiation, and embryo competence [38].

#### 2.2.3. Trace Minerals

Trace minerals act as enzymatic cofactors and modulators of redox balance, steroidogenesis, and reproductive signaling:

- **Zinc** is integral for meiotic spindle formation, chromatin condensation, and early embryonic gene transcription. Deficiency leads to increased aneuploidy, reduced cleavage rates, and poor blastocyst quality [39,40,41].
- **Selenium**, through glutathione peroxidase activity, protects oocytes and embryos from oxidative stress. Adequate selenium improves blastocyst cell numbers, enhances cryotolerance, and supports implantation success [37,38,41].
- **Copper and manganese** serve as cofactors for enzymes involved in steroidogenesis and connective tissue integrity. Deficiencies can impair luteal function, reduce progesterone synthesis, and compromise the uterine environment for early embryo survival [38,41,42].

## 3. Nutrition and Embryo Yield

Embryo yield in cattle reflects the cumulative efficiency of follicular recruitment, oocyte maturation, fertilization, early embryonic development, and post-transfer survival. Nutritional status influences each of these stages through coordinated endocrine, metabolic, and cellular mechanisms. The physiological effects of nutrition are therefore closely intertwined with assisted reproductive technology (ART) outcomes. Nutritionally optimized cows exhibit greater follicular responsiveness, increased ovulation rates, superior oocyte competence, and improved embryo survival following transfer [21,22,23,24,34,35]. In contrast, animals experiencing NEB or micronutrient deficiencies demonstrate attenuated superovulatory responses, reduced embryo recovery, and compromised blastocyst quality [24,34,35].

These observations underscore that embryo yield is not merely a function of hormonal stimulation, but also represents a metabolically programmed phenotype. Understanding how specific nutrients regulate the follicular microenvironment, oocyte bioenergetics, and embryonic resilience allows embryologists and veterinarians to integrate nutritional strategies with reproductive management to maximize donor potential [1,21,22,23,24,34,35].

### 3.1. Mechanistic Basis of Nutrient Effects on Embryo Yield

The influence of nutrition on embryo production extends beyond caloric adequacy. Specific nutrients act as

- Regulators of insulin/IGF-1–mediated follicular recruitment;
- Substrates for cellular metabolism and mitochondrial ATP production;
- Cofactors for steroidogenic and antioxidant enzymes;
- Modulators of oxidative stress;
- Donors for epigenetic methylation reactions.

Through these mechanisms, nutrition determines oocyte developmental competence and early embryonic viability [4,18,19,26,36,37]. Optimizing embryo yield therefore requires coordinated management of both macronutrients and micronutrients.

### 3.2. Effects of Specific Nutrients on Embryo Yield

#### 3.2.1. Energy and Protein

Energy availability regulates insulin and IGF-1 signaling, which stimulate granulosa cell proliferation and steroidogenesis, thereby enhancing follicular recruitment and oocyte maturation [4,34,35]. Adequate energy balance supports embryonic survival and blastocyst formation, whereas NEB disrupts endocrine signaling and reduces embryo yield [21,22,23,24].

Protein contributes essential amino acids required for cellular metabolism, mitochondrial function, and epigenetic programming [24]. Balanced protein intake improves oocyte competence and embryonic survival, while excessive degradable protein may impair the follicular environment through elevated blood urea nitrogen [24,34,35].

#### 3.2.2. Vitamins

Vitamins play regulatory roles in cytoplasmic maturation and oxidative defense:

- Vitamin A, via retinoic acid–mediated gene transcription, enhances oocyte cytoplasmic maturation and developmental competence [36].
- Vitamin E acts as a lipid-soluble antioxidant, reducing oxidative damage in oocytes and embryos and improving blastocyst viability [17,37].

These vitamins contribute directly to cellular integrity during oogenesis and early embryogenesis, thereby increasing embryo yield.

#### 3.2.3. Trace Minerals

Trace minerals function as enzymatic cofactors and redox stabilizers [38,39,40,41,42]:

- Selenium enhances glutathione peroxidase (GPx) activity, which improves redox balance, embryo quality, and cryotolerance [17,37], thereby increasing the yield of transferable embryos.
- Zinc plays an important role in maintaining meiotic spindle integrity and genomic stability, thereby reducing aneuploidy and improving embryo quality [37,40].

However, responses are dose dependent. While optimal supplementation supports steroidogenesis and antioxidant defenses, excessive intake may disrupt redox-sensitive signaling pathways and impair follicular maturation [43,44,45,46]. These findings highlight the need for precision-based supplementation strategies.

#### 3.2.4. Fatty Acids and Lipid Metabolism

Dietary fatty acids influence oocyte cytoplasmic maturation, mitochondrial efficiency, membrane composition, and prostaglandin synthesis—key determinants of follicular health and embryo development.

- Omega-3 PUFAs (EPA, DHA) improve mitochondrial bioenergetics, enhance oocyte cytoplasmic quality, and modulate prostaglandin pathways supporting luteal function and uterine receptivity. Supplementation increases blastocyst yield, improves post-thaw survival, and may reduce early embryonic loss [16,47,48].
- Balanced omega-6:omega-3 ratios prevent excessive pro-inflammatory signaling that could compromise early embryogenesis. Lipid composition also influences membrane fluidity, intracellular signaling, and oxidative resilience of developing embryos [16,47,48].

It is important to note that responses to omega-3 supplementation are variable. Differences in dosage, basal diet composition, production system, breed (dairy vs. beef), and environmental conditions contribute to inconsistent outcomes [47,48]. These discrepancies emphasize that nutritional interventions must be context-specific rather than universally applied.

#### 3.2.5. Oxidative Stress as a Convergent Mechanism

Oxidative stress represents a common pathway linking nutritional imbalance to reduced embryo yield. Reactive oxygen species (ROS) generated during metabolic stress can damage mitochondrial membranes, disrupt spindle formation, induce DNA fragmentation, and increase apoptotic signaling within embryos [49,50,51,52].

Antioxidant nutrients—including vitamin E, selenium, and zinc—mitigate these effects by stabilizing redox balance, enhancing blastocyst cell number, and improving cryotolerance. Thus, oxidative regulation is a central mediator through which micronutrients influence embryo survival and post-transfer pregnancy success.

### 3.3. Nutrients and Mechanisms Affecting Embryo Yield

**Figure 1:**
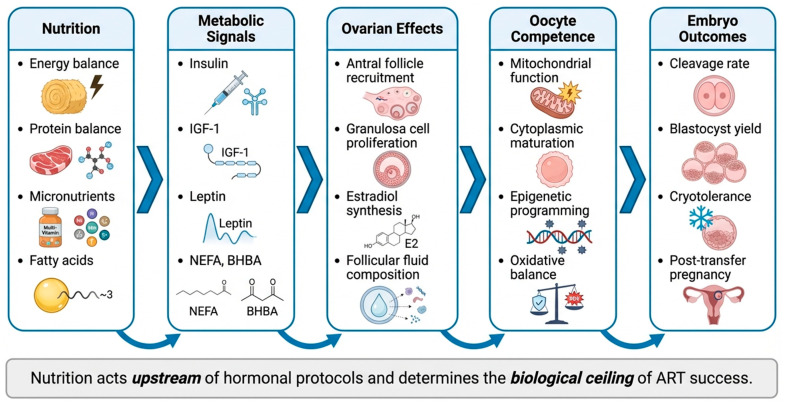
Nutritional regulation of embryo yield in cattle.

Schematic representation of the hierarchical relationship between nutrition, metabolic signaling, ovarian function, oocyte competence, and embryo yield in cattle (Figure 1). Adequate intake of energy, protein, micronutrients, and fatty acids regulates key metabolic hormones including insulin, IGF-1, and leptin. These signals modulate follicular recruitment, granulosa cell proliferation, and steroidogenesis, thereby influencing oocyte cytoplasmic and nuclear maturation. Optimized nutritional status improves cleavage rates, blastocyst yield, embryo cryotolerance, and post-transfer pregnancy outcomes, whereas nutritional deficits constrain the biological ceiling of ART success (Table 1).

**Key message:** Specific nutrients, including energy, protein, vitamins, trace minerals, and fatty acids, play mechanistic roles in regulating follicular development, oocyte competence, oxidative balance, and embryonic survival. Their integration into donor management programs is not merely supportive, but biologically essential for maximizing embryo yield, improving blastocyst quality, enhancing post-transfer pregnancy rates, and sustaining long-term reproductive efficiency in dairy and beef cattle [1,21,22,23,24,25,26,27,28].

## 4. Nutrition and Assisted Reproductive Technologies

**Figure 2:**
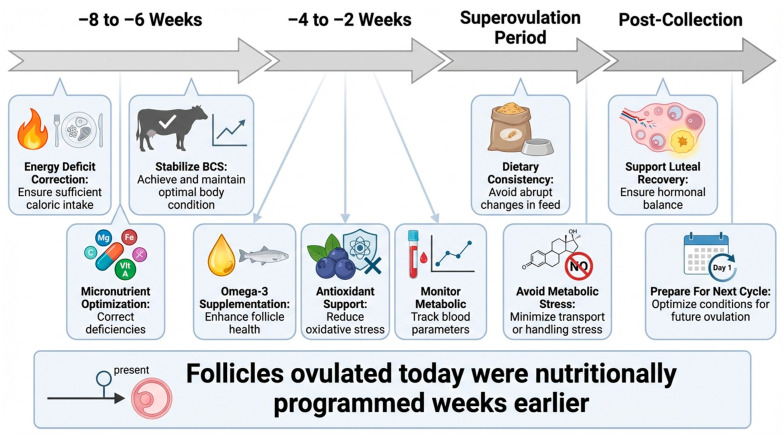
Temporal integration of nutritional management with superovulatory protocols.

Assisted reproductive technologies in cattle, including superovulation, ET, and IVP, rely heavily on the physiological and metabolic status of the donor. Nutrition acts as a primary modulator of follicular responsiveness, oocyte competence, embryo quality, and post-transfer survival, making it an integral component of ART program success [4,20,62]. Both macro- and micro-nutrient status, along with energy balance and body condition, influence hormonal signaling, the follicular microenvironments, and cellular pathways critical to embryo production [62,63]. Figure 2 presents conceptual framework illustrating the timing and coordination of nutritional interventions with superovulatory protocols.

Timeline depicting the strategic integration of nutritional management with superovulatory and embryo collection protocols in cattle. Nutritional optimization initiated 6–8 weeks prior to superovulation stabilizes energy balance, metabolic signaling, and follicular development. Targeted supplementation during follicular recruitment and gonadotropin stimulation supports oocyte competence and oxidative balance. Maintenance of dietary consistency during superovulation and post-collection nutritional support enhance embryo quality, cryotolerance, and donor recovery for subsequent ART cycles.

### 4.1. Superovulation Response and Follicular Dynamics

Superovulation protocols are designed to recruit and mature multiple follicles for oocyte collection. The efficacy of these protocols is tightly linked to donor nutrition. Energy balance, protein intake, and body condition score (BCS) regulate circulating concentrations of insulin, IGF-1, and leptin, which modulate FSH-mediated granulosa cell proliferation and estradiol synthesis [62,63]. Donors in NEB often exhibit reduced antral follicle populations and impaired steroidogenesis, leading to suboptimal ovulatory responses and lower oocyte yield [62,64]. Adequate energy and balanced protein intake restore metabolic signaling, ensuring greater follicular recruitment, increased ovulation rates, and improved superovulatory outcomes [64].

### 4.2. Oocyte Competence

Oocyte competence encompasses the ability of the oocyte to complete nuclear and cytoplasmic maturation, undergo fertilization, and support embryonic development. Nutrition influences oocyte competence at multiple levels:

- **Cytoplasmic maturation**: Adequate energy, protein, and micronutrients support mitochondrial function, ATP production, and organelle integrity [4,20,65].
- **Metabolic Cofactors in Bovine Oocyte Function**: In cattle, NAD^+^ availability has been increasingly recognized as a regulator of oocyte mitochondrial function and epigenetic remodeling through activation of sirtuin (SIRT) 1 and SIRT3, modulating oxidative phosphorylation and redox balance [66,67]. Supplementation with NAD^+^ precursors such as nicotinamide mononucleotide enhances mitochondrial efficiency and mitigates stress-related declines in oocyte competence. Similarly, α-ketoglutarate, a tricarboxylic acid cycle intermediate, supports DNA and histone demethylation, improving epigenetic stability and embryo developmental competence [68].
- **Oxidative balance**: Vitamins E and A, along with selenium, zinc, and copper, reduce reactive oxygen species (ROS)-induced damage, preserving spindle integrity and preventing DNA fragmentation [4,20,65].
- **Epigenetic programming**: Folate and B-vitamins supply methyl donors for DNA methylation and histone modifications, ensuring proper gene regulation during early embryogenesis [4,18,19,20].

Oocytes from nutritionally optimized donors demonstrate higher cleavage rates, improved blastocyst quality, and enhanced post-transfer survival, underscoring the mechanistic link between donor nutrition and embryo developmental potential [4,18,19,20,56].

### 4.3. Embryo Quality and Cryopreservation

Embryo quality is influenced by the nutrient-driven follicular microenvironment and oocyte health. Nutritionally optimized donors produce embryos with higher cell numbers, better inner cell mass development, and superior blastocyst morphology, enhancing implantation potential [4,20,59,65]. Lipid composition of oocytes, influenced by dietary fatty acids, affects membrane fluidity and cryotolerance. Omega-3 supplementation improves mitochondrial efficiency and reduces apoptosis in preimplantation embryos, resulting in higher survival rates after cryopreservation [59,65]. In contrast, metabolic stress, NEB, or micronutrient deficiencies can lead to lipid peroxidation, ROS accumulation, and compromised cryotolerance [4,20,49].

### 4.4. In Vitro Embryo Production (IVM/IVF/IVC) and Donor Nutrition

In vitro embryo production, encompassing oocyte retrieval, IVM, IVF, and IVC, has become a dominant platform in bovine ART. Although fertilization and early development occur outside the maternal tract, oocyte developmental competence remains fundamentally dependent on donor metabolic and nutritional status prior to collection.

Nutritional status influences

- Cumulus–oocyte complex quality;
- Cytoplasmic maturation;
- Mitochondrial function;
- Lipid accumulation and oxidative balance.

Oocytes recovered from donors in NEB exhibit altered mitochondrial membrane potential, increased lipid peroxidation, and reduced blastocyst rates following IVF. Conversely, optimized energy balance, antioxidant supplementation, and controlled fatty acid profiles improve IVP cleavage rates, blastocyst formation, and embryo cryotolerance.

Thus, even in IVP systems, donor nutrition sets the biological ceiling for embryo production efficiency.

### 4.5. Clinical Integration

Nutritional assessment and targeted interventions are as critical as hormonal management. Monitoring BCS, energy intake, protein balance, and micronutrient status prior to superovulation allows for the optimization of follicular response and oocyte competence [61,62]. Supplementation strategies, including antioxidants, trace minerals, and omega-3 fatty acids, should be aligned with the timing of follicular stimulation, oocyte retrieval, and ET [4,20,64,65]. By integrating nutritional management into ART protocols, clinicians can maximize embryo yield, improve blastocyst quality, enhance cryotolerance, and increase pregnancy rates, while reducing economic losses from suboptimal donor performance [4,20,61,62,63,64,65].

Key message: Nutrition is a foundational determinant of ART success. By modulating endocrine signaling, the follicular environment, oocyte competence, and embryo quality, dietary management directly affects superovulatory outcomes, blastocyst formation, and post-transfer survival. Veterinarians and embryologists should consider nutritional optimization an essential component of precision reproductive management, ensuring that metabolic and dietary factors support the biological potential of each donor cow for maximal ART efficiency [4,20,61,62,63,64,65].

## 5. Impact of Negative Energy Balance on Embryo Yield

Negative energy balance represents one of the most significant nutritional constraints to reproductive efficiency in modern cattle, particularly in high-producing dairy cows during early lactation and in underconditioned beef donors. Energy availability is the primary determinant of reproductive capacity. Negative energy balance, common in early lactation cows or underfed heifers, leads to reductions in circulating insulin, IGF-1, and leptin, which are key metabolic signals regulating GnRH pulsatility and subsequent FSH and LH release [21,22]. NEB occurs when energy expenditure exceeds dietary intake, resulting in mobilization of body reserves and profound alterations in metabolic and endocrine signaling. These changes have direct and indirect effects HPG axis function, follicular dynamics, oocyte competence, and early embryonic development, making NEB a critical determinant of embryo yield and ART success [69,70,71].

### 5.1. Disruption of the HPG Axis and Gonadotropin Secretion

Energy deficiency alters central reproductive control through reductions in circulating insulin, IGF-1, and leptin, which act as metabolic signals to the hypothalamus. Decreased leptin and insulin attenuate GnRH pulse frequency and amplitude, resulting in diminished pituitary secretion of FSH and LH [21,22,69,71]. This disruption compromises follicular recruitment and selection, leading to reduced antral follicle numbers and impaired ovulatory response [71]. In the context of superovulation, NEB donors often exhibit attenuated responsiveness to exogenous FSH, producing fewer ovulated follicles and a reduced number of recoverable oocytes or embryos [71].

### 5.2. Altered Follicular Microenvironment and Steroidogenesis

NEB negatively affects ovarian steroidogenesis by limiting granulosa cell proliferation and estradiol synthesis. Reduced IGF-1 signaling impairs FSH-mediated aromatase activity, resulting in suboptimal estradiol concentrations within developing follicles. This hormonal imbalance compromises follicular dominance, oocyte maturation, and luteal function, ultimately reducing embryo yield [69,72]. Additionally, increased circulating non-esterified fatty acids (NEFAs) and ketone bodies during NEB infiltrate follicular fluid, altering its biochemical composition and exerting lipotoxic effects on both granulosa cells and oocytes [69,73].

### 5.3. Compromised Oocyte Competence and Metabolic Stress

Oocyte developmental competence is particularly sensitive to energy availability. NEB reduces intracellular glucose and pyruvate availability, limiting ATP production and mitochondrial function within the oocyte [69,71]. Mitochondrial dysfunction impairs cytoplasmic maturation, spindle formation, and chromosomal segregation, increasing the risk of abnormal fertilization, poor cleavage rates, and developmental arrest [36,49]. At the molecular level, NEB is associated with altered expression of genes involved in oxidative phosphorylation, lipid metabolism, and stress response, resulting in embryos with reduced developmental potential [74].

Single-cell sequencing studies have advanced our understanding of how mitochondrial metabolism influences oocyte quality and subsequent embryo yield. Transcriptomic profiling at single-cell resolution reveals heterogeneity in mitochondrial gene expression, oxidative phosphorylation pathways, and metabolic stress responses among individual oocytes. High-quality oocytes typically exhibit coordinated regulation of mitochondrial biogenesis, ATP production, and redox balance, supporting developmental competence. In contrast, metabolically compromised oocytes show dysregulated energy pathways and oxidative stress signatures, which are associated with reduced fertilization rates, impaired embryo development, and lower overall embryo yield.

### 5.4. Gut–Ovary Axis and Bovine Reproductive Health

Emerging evidence indicates a gut–ovary axis in livestock whereby rumen microbiota composition influences systemic metabolites, inflammatory mediators, and endocrine signaling that may affect reproductive performance [75]. Microbiome-derived metabolites such as short-chain fatty acids (SCFAs) are produced by rumen fermentation and have been linked to host metabolic regulation and physiological interactions beyond digestion in ruminants [76]. While direct bovine studies on SCFAs and follicular microenvironment are limited, analogous mammalian research demonstrates gut microbiota-derived SCFAs promoting follicular maturation via endocrine modulation, suggesting similar mechanisms could influence bovine oocyte competence and embryo yield [77].

### 5.5. Oxidative Stress and Embryo Viability

**Figure 3:**
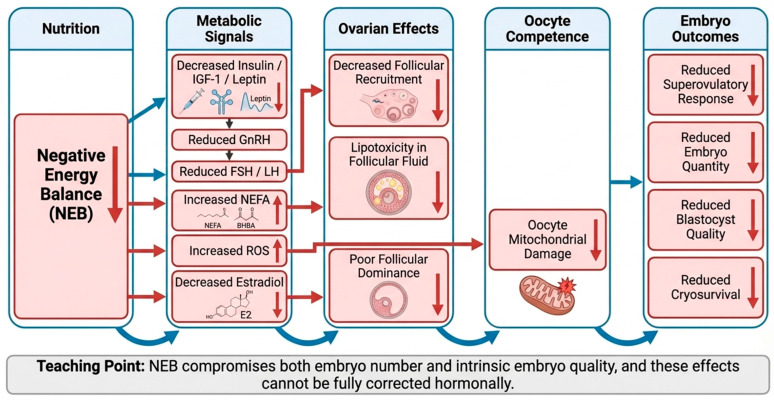
Mechanistic impact of negative energy balance on ART outcomes. ↑ increase, ↓ decrease.

NEB is characterized by increased oxidative stress due to excessive mobilization of adipose tissue and reduced antioxidant capacity. Elevated reactive oxygen species (ROS) damage cellular membranes, DNA, and mitochondria in oocytes and early embryos [52,58]. Oxidative stress compromises blastocyst cell number, increases apoptosis, and reduces cryotolerance, leading to higher rates of preimplantation embryo loss [69,78]. In ART programs, embryos derived from NEB donors often demonstrate lower survival following cryopreservation and reduced pregnancy rates after transfer [70]. Figure 3 depicts the proposed mechanisms linking negative energy balance to altered ART outcomes.

Pathophysiological pathways by which negative energy balance (NEB) impairs reproductive performance and embryo yield in cattle. NEB reduces circulating insulin, insulin-like growth factor (IGF)-1, and leptin concentrations, disrupting hypothalamic-pituitary-gonadal axis signaling and attenuating gonadotropin secretion. Concurrent increases in non-esterified fatty acids (NEFAs) and ketone bodies alter follicular fluid composition, induce lipotoxicity, and increase oxidative stress within oocytes. These changes result in impaired folliculogenesis, reduced oocyte developmental competence, decreased blastocyst quality, compromised cryotolerance, and lower pregnancy rates following embryo transfer.

### 5.6. Implications for ART Outcomes and Management

From an ART perspective, NEB negatively impacts every stage of embryo production, from follicular recruitment and oocyte quality to blastocyst viability and post-transfer survival. Donors in NEB consistently yield fewer embryos of inferior quality, increasing the cost per viable embryo and reducing program efficiency [69,70]. Effective management strategies include correcting energy deficits prior to superovulation, monitoring body condition score, and implementing energy-dense diets to stabilize metabolic status [55]. Supplementation with antioxidants, omega-3 fatty acids, and targeted micronutrients can partially mitigate oxidative stress but cannot fully compensate for severe energy deficiency [70,71].

**Key message:** The NEB exerts a multifaceted and deleterious effect on embryo yield through central endocrine disruption, altered follicular environments, compromised oocyte competence, and increased oxidative stress. Proactive management of energy balance is essential to optimize donor performance, maximize embryo quantity and quality, and ensure the biological and economic success of ART programs [21,22,23,24,69,70,71,72,73].

## 6. Metabolic Signaling and Precision Nutrition in Bovine ART

Reproductive efficiency in cattle can be conceptualized within a unified mechanistic framework in which intra-ovarian signaling, mitochondrial bioenergetics, and epigenetic stability are tightly regulated by nutritional inputs. Systemic energy balance reflects overall nutrient availability, but nutrient composition determines how ovarian cells interpret that status through conserved metabolic sensors, AMP-activated protein kinase (AMPK), mechanistic target of rapamycin (mTOR), and SIRT family which collectively integrate substrate supply with cellular growth, redox homeostasis, and mitochondrial function [4,79,80]. During NEB, decreased glucose, insulin, and IGF-1 increase the AMP:ATP ratio, activating AMPK. Activated AMPK suppresses mTORC1 signaling, shifting granulosa and cumulus cells toward a catabolic state characterized by reduced protein synthesis, diminished aromatase expression, impaired estradiol production, and limited follicular proliferation [81,82]. This metabolic restraint compromises oocyte cytoplasmic maturation and reduces developmental competence. In contrast, restoration of positive energy balance activates PI3K–Akt–mTOR signaling, promoting ribosomal biogenesis, mitochondrial expansion, and granulosa cell proliferation, thereby enhancing follicular recruitment and ovulatory responsiveness during superovulation [4,83]. Thus, the AMPK–mTOR axis functions as a metabolic rheostat that determines whether ovarian tissue prioritizes survival or reproduction.

Macronutrients modulate these pathways with distinct mechanistic signatures. Essential amino acids, particularly leucine, directly activate mTORC1, stimulating translational capacity and mitochondrial biogenesis in granulosa cells while supporting cumulus expansion and oocyte growth. Amino acids also fuel one-carbon metabolism; methionine-derived S-adenosylmethionine provides methyl donors for DNA and histone methylation during oocyte maturation and early embryogenesis, linking protein nutrition to epigenetic stability [84]. However, excessive rumen degradable protein elevates blood urea nitrogen, potentially disrupting follicular fluid homeostasis and oxidative balance [85]. Carbohydrate availability regulates insulin secretion and glycolytic flux within the cumulus–oocyte complex, where cumulus-derived pyruvate sustains oocyte oxidative phosphorylation [86,87]. Reduced glucogenic supply lowers NAD^+^ availability, attenuating SIRT1 and SIRT3 activity—key NAD^+^-dependent deacetylases governing mitochondrial biogenesis (via PGC-1α) and antioxidant defense (e.g., SOD2) [83]. Impaired SIRT signaling increases oxidative stress, disrupts spindle integrity, and compromises embryo viability. Fatty acids further influence mitochondrial efficiency and inflammatory tone: elevated NEFAs during NEB induce lipotoxic stress and excessive AMPK activation, whereas balanced omega-3 supplementation improves membrane fluidity, modulates NF-κB signaling, and may enhance SIRT3-mediated antioxidant resilience, thereby supporting blastocyst survival [69,83].

Micronutrients reinforce this network by stabilizing redox and epigenetic systems. Selenium supports glutathione peroxidase activity, zinc preserves meiotic spindle integrity and DNA repair, and folate-dependent pathways sustain methyl donor availability for genomic programming during early embryogenesis [88,89,90]. Collectively, embryo yield emerges as a metabolic phenotype determined by coordinated regulation of AMPK (energy sensing), mTOR (anabolic growth), and SIRT pathways (mitochondrial and redox control). Under NEB, excessive AMPK activation suppresses mTOR, reduces IGF-1 signaling, increases oxidative stress, limits SIRT activity, and impairs mitochondrial ATP production, culminating in reduced blastocyst cell number, increased apoptosis, compromised cryotolerance, and lower pregnancy rates [69,83,91].

From a precision nutrition perspective, optimizing assisted reproductive technologies (ARTs) requires aligning dietary strategies with these molecular checkpoints. Nutritional management before superovulation or oocyte pickup should aim to restore endocrine–metabolic coupling, stabilize NAD^+^-dependent SIRT activity, and maintain controlled mTOR activation to preserve mitochondrial competence and epigenetic fidelity. In this framework, nutrition is not merely supportive but establishes the biological ceiling for both in vivo and in vitro embryo production, defining the upper limits of follicular responsiveness, oocyte competence, and post-transfer developmental success [82,83].

## 7. Long-Term Effects of Early-Life Nutrition on Reproduction

**Figure 4:**
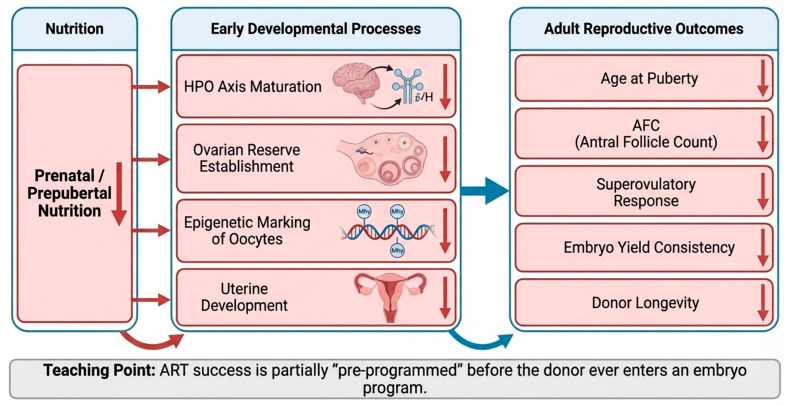
Long-term effects of early-life nutrition on reproductive potential and ART performance. ↓ decrease.

Early-life nutrition exerts profound and lasting effects on reproductive capacity in cattle through mechanisms collectively described as developmental programming (Figure 4). Nutritional status during critical windows—particularly the prenatal, neonatal, and prepubertal periods—influences the development of the hypothalamic–pituitary–ovarian (HPO) axis, ovarian reserve establishment, uterine development, and epigenetic regulation of gene expression [92,93,94]. These effects persist into adulthood and directly impact embryo yield, oocyte competence, and ART efficiency, making early-life nutrition a key determinant of long-term reproductive performance [92,95].

Conceptual model illustrating the influence of early-life nutrition on developmental programming of the hypothalamic-pituitary-ovarian axis, establishment of ovarian reserve, and epigenetic regulation of oocyte competence. Nutritional adequacy during prenatal, neonatal, and prepubertal periods supports proper gonadotropin signaling, primordial follicle survival, uterine development, and stable epigenetic marking. Nutritional insults during these critical windows result in reduced antral follicle counts, diminished superovulatory response, compromised embryo yield, and decreased reproductive longevity, with implications for lifetime ART efficiency.

### 7.1. Developmental Programming of the HPO Axis

The maturation of the HPO axis is highly sensitive to nutrient availability during early life. Adequate energy, protein, and micronutrient intake are required for proper hypothalamic development, GnRH neuron maturation, and pituitary gonadotrope differentiation [93,94]. Calves or heifers subjected to nutritional restrictions during this period often exhibit delayed puberty, reduced GnRH pulsatility, and impaired gonadotropin secretion, leading to suboptimal follicular recruitment later in life [93,96]. In contrast, heifers receiving optimal nutrition demonstrate earlier puberty onset, increased antral follicle counts, and enhanced ovarian responsiveness to both endogenous and exogenous gonadotropins-traits that are highly advantageous in ART donor candidates [92,96].

### 7.2. Establishment and Preservation of Ovarian Reserve

The ovarian reserve, defined by the number of primordial follicles established during fetal and early postnatal life, is a finite resource that determines lifelong reproductive potential. Undernutrition during fetal development or early growth can reduce primordial follicle numbers through increased apoptosis or impaired follicle formation [94,96]. A diminished ovarian reserve manifests later as lower antral follicle counts, reduced superovulatory response, and decreased embryo yield. Conversely, adequate early-life nutrition supports follicle survival, enhances vascularization of ovarian tissue, and promotes a robust follicular pool capable of sustaining repeated ART cycles [92,94].

### 7.3. Epigenetic Mechanisms and Oocyte Competence

One of the most significant consequences of early-life nutrition is its influence on epigenetic programming, including DNA methylation, histone modification, and non-coding RNA expression [92,97]. These epigenetic modifications regulate gene expression patterns critical for oocyte maturation, embryo development, and placental function [45,50]. Nutrients such as folate, methionine, choline, and B-vitamins provide methyl donors necessary for proper DNA methylation. Deficiencies during early development can lead to aberrant epigenetic marks in germ cells, resulting in oocytes with compromised developmental competence and embryos with altered growth trajectories [92,97].

From an ART perspective, epigenetically compromised oocytes may fertilize normally, but exhibit reduced blastocyst formation, impaired cryotolerance, and lower post-transfer survival. Moreover, altered epigenetic programming may influence offspring performance, raising concerns about transgenerational effects of poor early nutrition [78,95,97].

### 7.4. Uterine Development and Reproductive Longevity

Early-life nutrition also affects uterine growth, endometrial gland development, and vascularization [61,63]. Suboptimal nutrition can limit uterine capacity, reducing embryo implantation success and increasing early embryonic loss. Animals with compromised uterine development may exhibit a shortened reproductive lifespan, increased culling rates, and reduced lifetime productivity—factors that indirectly reduce the long-term efficiency of ART programs [94,96].

### 7.5. Implications for ART Donor Selection and Management

Recognizing the long-term impact of early-life nutrition is essential when selecting and managing donors. Heifers with documented adequate growth rates, optimal body conditions, and early puberty onset are more likely to demonstrate a robust ovarian reserve, superior oocyte competence, and consistent embryo yield [59,60]. Nutritional management strategies aimed at optimizing early growth should therefore be viewed as a long-term investment in ART success [92,94,96,97].

Table 2 shows the effects of early-life nutritional status, adult reproductive outcome, and their implications for ART outcome.

**Key message:** Early-life nutrition shapes reproductive capacity through developmental programming of the HPO axis, establishment of ovarian reserve, and epigenetic regulation of gene expression. These long-lasting effects influence embryo yield, quality, and ART outcomes well into adulthood [92,93,94,95,96,97]. For advanced reproductive programs, optimizing nutrition during early developmental windows is a critical strategy for maximizing donor potential, reproductive longevity, and genetic progress in cattle populations.

## 8. Practical Nutritional Strategies for ART Optimization

Optimizing nutritional management is a critical, controllable component of successful ART programs in cattle. Effective nutritional strategies must extend beyond generalized feeding recommendations and instead focus on individual donor assessment, precision supplementation, and strategic integration with hormonal superovulatory protocols. When appropriately implemented, these strategies enhance follicular responsiveness, oocyte competence, embryo quality, and post-transfer pregnancy outcomes while reducing variability in ART performance [1,2,37,39,47].

### 8.1. Donor Assessment and Nutritional Baseline Evaluation

The foundation of nutritional optimization begins with thorough donor evaluation. BCS remains a practical and reliable indicator of energy balance and metabolic status, with an optimal target range of 2.75–3.25 on a 5-point scale for ART donors [4]. Animals below this range often exhibit reduced ovarian responsiveness and poor oocyte quality, while overconditioned donors may suffer from insulin resistance and altered follicular steroidogenesis [63,107].

Beyond BCS, advanced ART programs increasingly incorporate metabolic profiling, including measurements of NEFAs, β-hydroxybutyrate (BHBA), glucose, insulin, and IGF-1. These indicators provide insight into energy balance and metabolic stress that may not be evident from physical assessment alone [2]. Evaluating dietary protein balance through blood urea nitrogen (BUN) is also critical, as elevated BUN is associated with compromised oocyte competence and early embryonic development [108,109]. Metabolic indicators and an ART risk assessment are given in Table 3.

### 8.2. Precision Supplementation of Macro- and Micronutrients

Once baseline status is established, targeted nutritional interventions can be implemented. Energy supplementation should aim to correct deficits gradually, avoiding abrupt dietary changes that may disrupt rumen function or exacerbate metabolic stress. Energy-dense feeds, protected fats, and controlled starch inclusion can help stabilize insulin and IGF-1 signaling prior to superovulation [35].

Protein nutrition should prioritize balanced rumen-degradable and undegradable protein to support amino acid availability while minimizing excess nitrogen excretion. Precision feeding reduces the risk of elevated BUN and maintains a favorable uterine and follicular environment for embryo development [107].

Micronutrient supplementation is particularly critical for ART donors. Trace minerals such as selenium, zinc, copper, and manganese, along with vitamins A, E, and B-complex vitamins, should be provided at levels that support antioxidant defense, steroidogenesis, and epigenetic regulation [119]. Organic or chelated mineral sources may improve bioavailability, especially in high-producing or metabolically stressed animals. Omega-3 fatty acid supplementation can further enhance oocyte cytoplasmic maturation, luteal function, blastocyst quality and embryo cryotolerance [120].

### 8.3. Precision Nutrition and Metabolomics

Integration of metabolomic profiling with reproductive performance data enables precision feeding strategies tailored to individual bovine donors by identifying metabolic predictors of reproductive outcomes. Untargeted metabolomics of bovine follicular fluid has revealed that amino acids, lipids, and other small-molecule metabolites vary with follicle development and are linked to oocyte maturation and competence [121,122]. Branched-chain amino acids such as glutamine in follicular fluid correlate with blastocyst formation rates in cattle [123]. Moreover, differential serum and follicular lipid metabolites, including phosphatidylcholines and triacylglycerols, have been associated with superovulatory response and embryo yield, supporting the use of metabolic biomarkers in precision reproductive nutrition and donor selection [122,123,124].

In *Drosophila melanogaster*, nutrient-sensing pathways such as GCN2, which detects amino acid deficiency, regulate oocyte growth, meiotic progression, and egg provisioning by modulating translation and stress-response signaling [125]. These pathways are highly conserved across species, including mammals and cattle. In bovine oocytes, amino acid availability similarly influences mTOR signaling and integrated stress response pathways, affecting protein synthesis, mitochondrial function, and redox balance, key determinants of oocyte competence and embryo development. Understanding GCN2-mediated nutrient sensing in flies provides mechanistic insight into how amino acid fluctuations and dietary protein balance may impact follicular maturation, oocyte quality, and subsequent embryo yield in cattle, supporting the development of precision nutritional strategies in reproductive management.

### 8.4. Integration with Superovulatory and ART Protocols

Nutritional interventions must be synchronized with reproductive management to maximize their effectiveness. Ideally, nutritional optimization begins 6–8 weeks prior to superovulation, allowing sufficient time for metabolic stabilization and follicular turnover [2]. This window is critical, as follicles destined for ovulation during superovulation are already undergoing development weeks earlier.

During superovulation, maintaining dietary consistency is essential to prevent fluctuations in metabolic hormones that could impair follicular response. Antioxidant supplementation during this period may mitigate oxidative stress induced by metabolic demands and hormonal stimulation [35,120]. Following embryo collection, continued nutritional support is important to promote uterine recovery, luteal function, and preparation for subsequent ART cycles.

### 8.5. Program-Level Benefits and Clinical Outcomes

When nutrition is fully integrated into ART protocols, donors consistently demonstrate improved superovulatory response, higher numbers of transferable embryos, enhanced blastocyst quality, and superior post-thaw survival [2,119,120]. These improvements reduce the cost per viable embryo, decrease the need for repeated hormonal stimulation, and improve overall program efficiency. Furthermore, optimized nutritional strategies support donor longevity, allowing for animals to participate in multiple ART cycles with sustained performance. Practical nutritional strategies to improve embryo technologies are given in Table 4.

**Key message:** Practical nutritional strategies for ART optimization require a precision-based, donor-specific approach that integrates metabolic assessment, targeted supplementation, and careful alignment with superovulatory protocols. Nutrition should be regarded as an essential component of advanced reproductive management—one that directly enhances embryo yield, ART efficiency, and long-term genetic progress in cattle.

## 9. Economic and Programmatic Implications

In advanced ART programs, economic sustainability is intrinsically linked to biological efficiency. Nutritional management exerts a powerful influence on ART outcomes by determining embryo yield, embryo quality, donor longevity, and post-transfer pregnancy success, all of which directly affect program costs and returns [3,129,130,131,132,133,134]. For veterinarians, geneticists and embryologists overseeing high-value genetic programs, the economic implications of nutrition extend beyond feed costs to encompass efficiency of hormonal protocols, labor utilization, embryo utilization rates, and long-term herd genetic gain [91,92,93,94,95,96].

### 9.1. Embryo Yield and Cost per Transferable Embryo

Embryo yield is a primary driver of ART economics. Nutritionally optimized donors consistently produce higher numbers of transferable embryos per collection, reducing the cost per viable embryo [3,132]. Poor nutritional status, particularly negative energy balance or micronutrient deficiencies, results in suboptimal superovulatory response, increased numbers of unfertilized ova or degenerate embryos, and greater variability in outcomes [4,35,36,37,38,39,40,41,42,130]. Each failed or low-yield collection represents sunk costs in gonadotropins, synchronization agents, labor, and veterinary services. By contrast, targeted nutritional management improves follicular recruitment and oocyte competence, increasing the proportion of embryos reaching transferable or freezable quality grades [3,4,130].

### 9.2. Embryo Quality, Cryotolerance, and Utilization Efficiency

Embryo quality directly influences downstream costs and success rates. High-quality blastocysts demonstrate superior cryotolerance, post-thaw survival, and pregnancy rates following transfer, maximizing the return on investment from each ART cycle [97,98,99,100]. Nutritionally compromised donors often produce embryos with reduced cell numbers, higher apoptotic indices, and poor membrane integrity, leading to increased embryo loss during cryopreservation and after transfer [3,135,136,137,138]. The economic consequence is not only wasted embryos but also increased recipient preparation costs and lower overall pregnancy efficiency. Nutritional optimization enhances embryo robustness, improving utilization rates and reducing the need for repeated recipient synchronization [135,136,137,138,139,140,141].

### 9.3. Donor Longevity and Program Stability

From a programmatic standpoint, donor longevity is a critical economic consideration. Donors experiencing chronic metabolic stress, repeated negative energy balance, or inadequate micronutrient support are more prone to reproductive fatigue, declining embryo yield, metabolic disorders, and early culling [129,134,135,136,137]. Replacing elite donors entails significant genetic, financial, and time-related costs. Nutritional strategies that support metabolic health and reproductive resilience allow donors to participate in multiple ART cycles with consistent performance, stabilizing program output and protecting long-term genetic investments [129,130,131,135,136,137].

### 9.4. Integration with Genetic Gain and Strategic Breeding Goals

Advanced ART operations are often embedded within broader genetic improvement strategies. Enhanced embryo yield and quality accelerate genetic dissemination, enabling greater selection intensity and faster generation turnover [131,134]. Nutritional optimization indirectly amplifies genetic progress by increasing the number of high-quality embryos available from elite donors and improving pregnancy success rates in recipients [3,131,132,133,134,136,137,138,139,140,141]. Conversely, nutritional neglect undermines genetic potential by limiting the biological expression of superior genetics through reduced embryo production and survival [136,137,138,139,140,141].

### 9.5. Cost–Benefit Perspective of Nutritional Investment

While targeted nutritional interventions (Table 5) incur direct costs, such as premium feeds, precision supplementation, and metabolic monitoring, these expenses are typically marginal relative to the costs of failed ART cycles or lost genetic opportunity [142,143,144].

Studies consistently demonstrate that investment in nutritional optimization yields favorable cost–benefit ratios, particularly in high-value donor programs [1,142,143,144]. Improved embryo yield, reduced hormonal waste, higher pregnancy rates, and extended donor lifespan collectively outweigh the incremental costs of enhanced nutritional management. A cost–benefit summary for ART programs is presented in Table 6.

### 9.6. Program-Level Decision-Making

Economic decision-making must incorporate both short-term ART efficiency and long-term program sustainability [151]. Nutrition should be viewed not as an ancillary expense, but as a strategic investment that underpins biological performance and financial viability [151]. Integrating nutritional metrics into ART performance evaluation enables data-driven adjustments that enhance consistency, predictability, and profitability [138,139,140,141,152,153].

**Key message:** The economic and programmatic implications of nutrition in advanced ART operations are substantial. By directly influencing embryo yield, quality, utilization efficiency, and donor longevity, nutritional management serves as a critical lever for optimizing both biological outcomes and economic returns. Precision nutrition represents a cornerstone of sustainable, high-performance ART programs and long-term genetic advancement in cattle.

## 10. Future Directions

To advance precision reproductive nutrition and meet the expectations of evidence-based practices, future research should move beyond associative observations and adopt mechanistically driven, context-specific experimental designs. The following priorities are proposed:

### 10.1. Mechanistic Integration of Nutrient–Signaling Pathways

Greater emphasis is needed on delineating how nutrients regulate intracellular metabolic sensors, including AMPK, mTOR, and SIRT pathways, within granulosa cells, cumulus–oocyte complexes, and early embryos. Clarifying how energy balance, amino acids, and fatty acids influence mitochondrial biogenesis, redox homeostasis, autophagy, and epigenetic programming will provide causal links between diet and embryo competence. Multi-omics approaches (transcriptomics, metabolomics, lipidomics, and epigenomics) integrated with ART outcomes should be prioritized to identify predictive biomarkers of oocyte and embryo quality.

### 10.2. Dose–Response and Threshold Studies

Many micronutrients (e.g., selenium, zinc, omega-3 fatty acids) exhibit narrow optimal ranges, with both deficiency and excess impairing embryo development. Rigorous dose–response trials under controlled production conditions are required to define physiological thresholds, redox-sensitive tipping points, and interactions with basal diet composition. Such studies will reduce variability observed across dairy and beef systems.

### 10.3. Precision Nutrition for ART Donors

Future work should develop metabolic phenotyping tools to stratify donor cows based on body condition score, insulin sensitivity, NEFA/BHBA profiles, and inflammatory markers prior to superovulation. Tailored nutritional interventions—rather than uniform supplementation strategies—may enhance follicular responsiveness, oocyte competence, and blastocyst yield while minimizing metabolic stress.

### 10.4. Bridging In Vitro and In Vivo Systems

Caution remains warranted when extrapolating in vitro findings to in vivo embryo production. Parallel studies comparing nutritional modulation effects across ovum pick-up-IVP and multiple ovulation embryo transfer systems are needed to determine whether cellular mechanisms identified in vitro translate into improved pregnancy and calving outcomes.

### 10.5. Long-Term Developmental and Epigenetic Consequences

Emerging evidence suggests that donor nutrition may influence embryonic epigenetic programming and potentially offspring performance. Longitudinal studies evaluating post-transfer pregnancy maintenance, calf health, growth, and reproductive performance will clarify whether nutritional optimization at the donor stage yields transgenerational benefits.

### 10.6. Standardization and Reporting Frameworks

To enhance reproducibility, future studies should adopt standardized reporting of diet composition, micronutrient bioavailability, metabolic status, and ART protocols. Harmonized methodologies will facilitate meta-analyses and strengthen translational recommendations for commercial embryo production systems.

### 10.7. Overall Perspective

Future investigations should integrate molecular biology, reproductive physiology, and controlled nutritional management to define actionable thresholds that optimize embryo yield without inducing metabolic perturbations. Advancing from descriptive correlations to mechanistic precision will enable nutrition to be strategically positioned as a core determinant—rather than a supportive factor—of ART efficiency in dairy and beef cattle.

## 11. Conclusions

Nutrition is a foundational determinant of embryo production efficiency in cattle. Both in vivo and in vitro embryo systems rely on adequate energy balance, macronutrient intake, micronutrient status, and metabolic stability to support follicular development, oocyte competence, and embryo survival. Negative energy balance, oxidative stress, and micronutrient deficiencies limit the biological potential of ART outcomes. Precision nutritional management, implemented before and during ART cycles, enhances superovulatory response, blastocyst quality, cryotolerance, and pregnancy rates, while also improving economic efficiency and donor longevity. Early-life nutritional programming further influences lifetime ART potential by shaping ovarian reserve and reproductive capacity. Integrating metabolic monitoring, targeted supplementation, and synchronized feeding strategies into reproductive protocols is therefore critical for maximizing embryo yield and sustaining high-performance cattle breeding systems.

## Figures and Tables

**Table 1:** Nutrients and Their Mechanisms on Embryo Yield.

Nutrient	Primary Mechanism	Effect on Embryo Yield	References
Energy	Insulin/IGF-1 signaling improving follicular and oocyte development	↑ Follicular recruitment & embryonic survival	[27,28,53,54]
Protein	Amino acid availability supporting cellular metabolism	↑ Oocyte competence & embryonic survival	[55,56]
Vitamin A	Retinoic acid–mediated gene transcription during oocyte maturation	↑ Cytoplasmic maturation	[36,57]
Vitamin E	Antioxidant reducing oxidative stress in oocytes/embryos	↑ Blastocyst viability	[17,49,50]
Selenium	Enhances glutathione peroxidase (GPx) activity & redox balance	↑ Cryotolerance & embryo quality	[17,37,43,44,51,52]
Zinc	Promotes meiotic spindle integrity & genomic stability	↓ Aneuploidy & ↑ embryo quality	[37,40,43,45,46,58]
Omega-3 FA	Modulates membrane fluidity & mitochondrial efficiency	↑ Blastocyst survival & embryo development	[59,60,61]

↑, increase; ↓, decrease.

**Table 2:** Effects of Early-Life Nutrition on Adult ART Performance.

Early-Life Status	Adult Outcome	ART Implication	References
Undernutrition	↓ ovarian reserve	↓ embryo yield	[98,99]
Delayed puberty	↓ AFC	Variable response	[100,101]
Poor micronutrients	Epigenetic errors	↓ embryo quality	[102,103]
Optimal growth	Robust follicular pool	Consistent high yield	[104,105,106]

↓ decrease.

**Table 3:** Metabolic Indicators and ART Risk Assessment.

Parameter	Optimal Range	ART Risk If Abnormal	References
BCS	2.75–3.25	Poor ovarian response	[110,111]
NEFA & BHBA	Low	Lipotoxic oocytes; reduced cleavage	[112,113,114,115]
Insulin	Adequate	Impaired folliculogenesis	[116]
IGF-1	Adequate	Reduced FSH response	[4,117]
BUN	Moderate	Embryo toxicity	[5,110,118]

**Table 4:** Nutritional Failures vs. Optimized Programs.

Parameter	Poor Nutrition	Optimized Nutrition	References
Embryos/flush	Low & variable	High & consistent	[126,127]
Transferable embryos	Reduced	Maximized	[128]
Cryosurvival	Poor	Excellent	[128]
Donor longevity	Shortened	Extended	[126]
Cost per embryo	High	Reduced	[62]

**Table 5:** Nutritional Intervention Timing Framework for ART Optimization.

Intervention Type	Timing	Target Nutrients	Rationale
Acute (1–2 weeks)	Peri-superovulatory	Vitamin E (1000–2000 IU/day), selenium (0.3 ppm diet), omega-3 FA (20–40 g protected source/day)	Reduce oxidative stress, improve cryotolerance
Intermediate (2–4 weeks)	Follicular wave recruitment	Vitamin A (50,000–80,000 IU/day), B-vitamins	Support cytoplasmic maturation & methylation
Chronic (6–8 weeks)	Pre-superovulation	Energy balance correction, BCS 2.75–3.25	Optimize follicular pool
Lifetime	Prenatal–prepubertal	Balanced growth (0.7–0.8 kg/day ADG)	Program ovarian reserve

**Table 6:** Cost–Benefit Summary for ART Programs.

Investment	Cost Impact	Economic Return	References
Precision feeding	Moderate cost	↓ Embryo cost/improved efficiency	[145,146]
Antioxidants	Low cost	↑ Embryo survival	[147,148,149]
Omega-3 FA	Moderate cost	↑ pregnancy rates	[48,59,150]
Metabolic monitoring	Low–moderate cost	↓ failed collections/early intervention	[54]
Early-life nutrition	Long-term investment	↑ lifetime ART output	[104,105,106,150]

↑, increase; ↓ decrease.

## Data Availability

No new data were created.
